# Ethnicity- and Sex-Based Discrimination and the Maintenance of Self-Esteem

**DOI:** 10.1371/journal.pone.0124622

**Published:** 2015-05-15

**Authors:** Jan-Erik Lönnqvist, Heike Hennig-Schmidt, Gari Walkowitz

**Affiliations:** 1 Swedish School of Social Science, University of Helsinki, Helsinki, Finland; 2 Laboratory for Experimental Economics, University of Bonn, Bonn, Germany, & University of Oslo, Oslo, Norway; 3 Department of Corporate Development and Business Ethics, University of Cologne, Cologne, Germany, & Laboratory for Experimental Economics, University of Bonn, Bonn, Germany; Middlesex University London, UNITED KINGDOM

## Abstract

The psychological underpinnings of labor market discrimination were investigated by having participants from Israel, the West Bank and Germany (*N* = 205) act as employers in a stylized employment task in which they ranked, set wages, and imposed a minimum effort level on applicants. State self-esteem was measured before and after the employment task, in which applicant ethnicity and sex were salient. The applicants were real people and all behavior was monetarily incentivized. Supporting the full self-esteem hypothesis of the social identity approach, low self-esteem in women was associated with assigning higher wages to women than to men, and such behavior was related to the maintenance of self-esteem. The narrower hypothesis that successful intergroup discrimination serves to protect self-esteem received broader support. Across all participants, both ethnicity- and sex-based discrimination of out-groups were associated with the maintenance of self-esteem, with the former showing a stronger association than the latter.

## Introduction

We begin by making two observations. On the one hand, the employer-applicant relationship may well be one of the most important and well-documented real-world contexts in which intergroup discrimination, especially with regard to ethnicity and sex, is known to be a major problem [1–6]. On the other hand, the self-esteem hypothesis of the currently highly influential social identity approach was formulated to explain intergroup discrimination—by establishing positive distinctness for the in-group, in-group members are establishing positive self-esteem for themselves. However, to the best of our knowledge, there appears to be no previous literature that directly addresses whether the self-esteem hypothesis could help explain discrimination by employers. The present research was designed to start filling this gap in our knowledge.

The lack of previous research on the self-esteem hypothesis in an employment context could be considered especially surprising as the social identity approach to intergroup relations has, during the last decades, emerged as a meta-theory for work on group processes. Core constructs of the theory, such as categorization, status, legitimacy, and identity, appear essential to our understanding of both interpersonal and intergroup relations. Furthermore, the self-esteem hypothesis can arguably be considered the most central, intuitive, and appealing aspect of the entire theory (but see also next section). One reason for this lack of research could be that the veracity of the self-esteem hypothesis is still, after more than 40 studies, to be verified [7]. However, as we will argue, much of the prior research on the hypothesis has been plagued by lack of realism and other methodological problems. The present study attempted to avoid the most common methodological pitfalls, and investigated the self-esteem hypothesis in a stylized employer-applicant relationship, in which participants from Israel, the West Bank, and Germany acted as employers that ranked, set wages, and imposed a minimum effort level on applicants whose ethnicity and sex were salient.

### The Self-Esteem Hypothesis

The term ‘self-esteem hypothesis’ was coined two decades ago by Abrams and Hogg [8] in their highly influential review of the social identity approach to intergroup discrimination. Based on the work of Tajfel and colleagues [9], [10], who had shown that participants divided into groups will discriminate against out-groups, Abrams and Hogg [8] identified self-esteem as the ex-ante assumed primary motivating force of intergroup discrimination. More precisely, they specified two corollaries from the notion that by establishing positive distinctness for the in-group, in-group members are establishing a positive social identity for themselves and thereby positive self-esteem (also [11]). These corollaries were (1) that successful intergroup discrimination elevates self-esteem, and (2) that depressed self-esteem promotes intergroup discrimination because of a need for self-esteem. Abrams and Hogg [8] found almost no empirical support for either corollary.

The self-esteem hypothesis has been criticized by some proponents of social identity theory. For instance, Turner [12] criticized corollary (2) for reducing social identity processes to a quest for self-esteem. In contrast, he claimed that the theory really is more about the interplay of social identity and social reality (e.g., stemming from social-structural variables such as group status, stability, legitimacy, permeability); self-esteem was only introduced to grapple with the operationalization of positive group distinctiveness [13]. Hogg [14] also somewhat revised his stance by dethroning self-esteem in favor of uncertainty reduction, making the latter the most basic motivation of in-group bias. But this shift in emphasis was not very radical—self-esteem was still part of the picture as a constituent of uncertainty reduction. More generally, we of course agree that social identity theory cannot be equated with any two corollaries. However, our aim was not to test social identity theory on a general level. Rather, we were interested in how social identity theory can help understand positive group-distinctiveness or in-group bias in a selection context. Despite some criticism of the self-esteem hypothesis, it should offer a viable means by which to empirically approach this question.

Later on, Rubin and Hewstone [7] reviewed the accumulated literature anew and found more than 40 studies in which either hypothesis had been tested. They found 18 studies supporting and 13 studies rejecting corollary (1), and seven studies supporting and 28 studies rejecting corollary (2). Only eight studies had tested both corollaries simultaneously (which, as Rubin and Hewstone [7] noted, is how the self-esteem hypothesis should be tested), and of these only one found support for both corollaries. They thus concluded their review by tentatively suggesting that intergroup discrimination leads to an increase in self-esteem, but that low self-esteem does not motivate intergroup discrimination. However, they also noted that some ambiguity remains due to the vast array of methods and experimental designs that have been used to test the self-esteem hypothesis, and ended their review by stating “further clarification is required in order to allow a more thorough investigation of the relation between self-esteem and intergroup discrimination” (p. 59). One noteworthy ambiguity was introduced by Lemyre and Smith [15], who based on their results in what was perhaps the most carefully conducted study on the self-esteem hypothesis, suggested that discrimination does not elevate self-esteem, but functions to maintain threatened self-esteem (for similar results, see [16], [17]). But this and other ambiguities were never resolved—in a more recent review, Hornsey [18] noted that “since the late 1990s, work on the ‘self-esteem hypothesis’ has fallen out of fashion” and that “many of the original architects of the social identity approach have recently fallen silent on the topic” (p. 214). The present study sought to reignite interest in the self-esteem hypothesis by providing a test of its explanatory power in a context in which real-life discrimination is a major problem.

### Minimal or Natural Groups

The majority of the empirical research on the social identity approach has been conducted using the minimal group paradigm. Minimal groups, in contrast to natural groups, are experimenter-created groups in which membership is determined by arbitrary and meaningless criteria (e.g., preference of one artist over another). The minimal group paradigm uses minimal groups to investigate the minimal conditions necessary for discrimination to occur among groups [9]. Although the social identity approach was developed to explain results obtained in research on minimal groups, the two cannot be equated. It seems possible that the implications of intergroup discrimination for self-esteem may be stronger when group boundaries are meaningful because such groups may suggest something about the qualities of the group members (one group may for instance be more prestigious than the other). Discrimination may thus be more strongly linked to self-esteem in a context of naturally occurring in-groups and out-groups, especially when the out-groups form a naturally occurring and salient threat to the self-concept [19], [20]. Supporting this line of reasoning is research that shows that the self-esteem hypothesis may only be applicable to individuals who identify with the in-group ([21], [22]; for an experimental manipulation of group identification, see [23]).

One of the reasons why natural groups have more seldom been the subject of study could be the distinction made by Turner [24] between normative and competitive discrimination. Normative discrimination is prescribed by previous intergroup relations, and is motivated primarily by a need to conform to perceived norms (e.g., paying men more than women), whereas competitive discrimination operates against intergroup norms to positively distinguish the in-group (e.g., paying women more than men). A conservative interpretation of social identity theory could suggest that only the latter should actually be related to an increase in self-esteem. However, normative and competitive discrimination may be extremely difficult to disentangle when doing research on naturally occurring rather than minimal groups. But this may not be a crucial problem in light of empirical evidence that suggests normative and competitive discrimination to have similar relations with self-esteem [7]. Relying on such evidence, and because our main concern was with real life discrimination, the present study, unlike most of the previous work within the social identity approach, used natural groups (ethnicity and sex) instead of minimal groups.

### Measuring Discrimination

One key issue in testing the self-esteem hypothesis has been how to measure discrimination. Studies conducted within the minimal group paradigm have typically derived indices of discrimination from the relative allocation of points to in-group and out-group. By contrast, studies conducted with natural groups have typically given the participants the opportunity to express their attitude towards the out-group, for instance by rating personality characteristics of the out-group [21], rating the competence of the out-group [25], or simply rating aggressive and benevolent attitudes concerning the out-group [26]. As noted by Abrams and Hogg [8], such studies may directly tap the esteem in which subjects hold their own group, and relating such measures to questionnaire measures of self-esteem may confound the results. Therefore, as Rubin and Hewstone [7] argued, more behavioral measures of discrimination should be given preference.

We sought to investigate discrimination in the natural context provided by the employer-applicant relationship. All participants reported on in this paper were assigned the role of an employer. The employment task consisted of rank-ordering the applicants, setting their wages, and optionally imposing a minimum effort level on them. All of these tasks provided distinct opportunities for ethnic or sex-based discrimination; i.e., participants could prefer to hire a compatriot (in the case of sex-discrimination, a same sex applicant), set higher wages for compatriots (same sex applicants), and impose, more frequently, a minimum effort level on foreigners (opposite sex applicants).

There are some important differences among the three discrimination indices. The rank-ordering task differed from the other tasks in that it forced the employer to favor either the in-group or the out-group; no totally neutral or impartial option was provided (one applicant had to be picked first). Discrimination could be more strongly related to elevated self-esteem in the absence of an impartial or totally fair option [27]. The wage payment and the imposition of a minimum effort level task provided an impartial option, but differed from each other by measuring discrimination in the positive (allocating money) and negative (forcing payback) domains, respectively (for a meta-analysis on positive-negative asymmetry in social discrimination, see [28]). This is important, as critiques have suggested that the processes identified by the social identity approach may only apply in the positive domain [29].

### Purpose of the Present Research

In sum, the present research sought to investigate whether the self-esteem hypothesis proposed by the social identity approach could help us understand ethnicity- and sex-based discrimination in the employer-applicant relationship. Based on corollary (1) of the self-esteem hypothesis, we thus hypothesized that successful intergroup discrimination would be associated with the elevation or maintenance of self-esteem (Hypothesis (1)), and, based on corollary (2), that depressed self-esteem would be related to a higher level of intergroup discrimination (Hypothesis (2)). The relevant group categorizations were ethnicity and sex, both of which were salient in the descriptions of the applicants. Ethnicity and sex are probably among the most important groups that people use to define their identity [30–32]. Furthermore, ethnicity- and sex-based discrimination in the labor market are among the most well-documented and important forms of prevailing discrimination [1], [33]. It can therefore be considered surprising that the explanatory power of the self-esteem hypothesis has not previously been put to test in this natural context. More specifically, we investigated the behavior that samples of Israeli, Palestinian, and German men and women express towards each other in an employment task. The complex relations among the three ethnic groups—returned to in the discussion—should make the mutual behaviors of these three groups especially interesting for applying psychological theory.

## Method

### Participants and Procedure

Students from various faculties in six distinct universities, two of them located in Israel (Beer-Sheva; Jerusalem), two in the West Bank (Abu-Dis; Ramallah) and two in Germany (Bonn; Cologne), were invited to take part in an incentivized decision experiment. The experimental sessions were conducted using pen and paper. Participants were seated in laboratory cubicles (Jerusalem; Bonn; Cologne) or large class rooms to secure anonymity. The experiment was run in each university with six groups of twelve participants, half of them acted as employers, the other half as applicants, in a stylized one-shot employer-applicant relationship. It is important to note that we had two universities each in Israel, the West Bank, and Germany to avoid in-group and discriminatory effects at the level of the university: applicants were always from a non-specified university different from that of the employers, and participants were made aware of this at the outset of the experimental sessions.

Only employers are reported on in the present study. At the outset, we had 72 employers from each region. After excluding those ten participants whose ethnicity did not match the location the participant was recruited in, as well as one Israeli participant with missing values, we were left with 68 Palestinian participants (34 women, mean age (*SD*) = 21.0 (2.0)), 68 Israeli participants (34 women, mean age (*SD*) = 24.9 (1.9)), and 69 German participants (34 Women, mean age (*SD*) = 24.3 (3.6)). Each participant generated one independent observation (employers did not, for instance, interact with each other, but made their decisions completely independently). In Germany, participants were recruited through the universities of Bonn and Cologne mailing lists to which they had signed up in order to take part in research conducted at the Laboratory of Experimental Economics Research. In Israel and the West Bank, participants were recruited through fliers posted on University notice boards.

Participants first completed a state self-esteem questionnaire. Each employer then received an identical set of twelve short résumés, each from a different applicant. Because the applicants were real people, we needed to pre-select them based on demographic data and school grades in order to be able to give employers real information about the applicants but still keep the set of twelve résumés identical. Three variables varied systematically in the résumés: Region (*Israel* or *West Bank* or *Germany*), Sex (*Men* or *Women)* and Additional Comments (*Applicant belongs to the best 50% of Israeli/Palestinian/German applicants with respect to school grades* or *none*), but only the first two were of interest for the present research. The application also contained three constant filler items: Age (*Between 18 and 29 years old*), Family status (*Single*), and Educational background (*General qualification for university entrance*). Employers first rank-ordered the résumés, and then decided which wage to pay each of the applicants in case of employment. In addition, participants had the opportunity to impose a fixed minimum effort level on each applicant. Immediately after completing the employment task, participants again completed the same state self-esteem questionnaire. Finally, participants answered some questionnaires not related to this study. All participants were paid a show-up fee of 20 NIS (Israel; West Bank); 4 Euro (Germany), as well as what they earned in the experiment. Employers’ earnings in the experiment were, as explained in detail below, determined by matching their decisions with those of the applicants (for computation of pay-offs, each employer was matched with one applicant, and they were paid according to their decisions; for details on the incentive compatible matching procedure, see Subsection ‘Rank-ordering of applicants‘). Employers earned on average 84.6 NIS in Israel, 67.4 NIS in the West Bank, and 16.9 Euro in Germany.

### Measures

#### Self-esteem questionnaire

The state self-esteem scale consisted of three items: “I feel good about myself”, “I feel confident that I understand things” and “I feel as smart as others” selected from the state self-esteem scale [34]. Each item was responded to on a scale from 1 (*not at all*) to 5 (*extremely*). The original English items were translated and back translated into Hebrew, Arabic, and German.

Table 1 shows the descriptive statistics of our self-esteem questionnaire. The means, varying between 3.37 and 3.74, were reasonably close to the mid-point (3.0) of the scale, indicating that ceiling or roof effects were not likely to have distorted the results. Although the reliabilities look rather normal for a three-item scale, they can be considered slightly low, and raise the possibility that measurement error may have attenuated some of the results we report. But low internal consistency reliabilities should not generally be interpreted as threatening measurement validity: McCrae, Kurtz, Yamagata and Terracciano [35] argued and showed that internal consistency reliability was not related to various validity criteria. However, the test retest reliability, which McCrae and colleagues [35] recommended to be used instead, was related to several validity criteria. In the present study, the test retest correlations in the samples of Israelis, Germans, and Palestinians were *r* = .66, *r* = .54, and *r* = .84, averaging a respectable *r* = .68. Nevertheless, because the internal consistency reliabilities were somewhat low, any results we may find should be considered all the more convincing considering the fair amount of measurement error in our dependent variable.

**Table 1:** Descriptive statistics for our questionnaire measure of self-esteem and pairwise t-test statistics for the difference in means between pre- and post-decision self-esteem.

	Pre-decision self-esteem		Post-decision self-esteem		
	*M (SD)*	α	*M (SD)*	α	*t*
**Israel (*N* = 68)**	3.51 (.53)	.38	3.37 (.54)	.43	3.20**
**West Bank (*N* = 68)**	3.73 (.58)	.62	3.71 (.58)	.57	0.35
**Germany (*N* = 69)**	3.55 (.61)	.49	3.40 (.67)	.46	2.13*

* *p* < .05,

** *p* < .01,

*** *p* < .001

#### Rank-ordering of applicants

Each participant was asked to rank-order the résumés by assigning a unique rank from one to twelve to each résumé. This rank-ordering influenced the probability of actual employment (a matching mechanism ensured that the higher an employer ranked the applicant, the higher was the probability of the applicant actually being hired to work for that employer). The matching of employers and applicants (necessary to compute payoffs) was executed at the very end of each session. From the rank-ordering of the twelve applicants, we coded whether the first choice that the employer made was a compatriot (of the same sex). We also ran all analyses using mean rankings, but because the results were virtually identical to those obtained when coding only the first choice, we present only the latter.

#### Wage payment to applicants

After setting a personal rank-order, employers chose a wage (10, 20, 30, 40, 50, 60, 70, 80, 90, 100, or 120 Points) to be paid to an applicant in case of employment. Because employers at this stage did not know which of the applicants they would actually employ, they determined a wage for all twelve applicants. At the second stage, conducted after all employer decisions had been collected, applicants decided on how much costly effort (0–120 Units) to exert (i.e., pay back) for each potential wage payment.

The income of one specific employer/applicant-dyad depended on the wage paid to that specific applicant and the effort exerted for that specific wage by the applicant. Employer’s payoff equaled twice the applicant’s effort minus the applicant’s wage. The applicant’s payoff was equal to the received wage minus the costs for the exerted effort (each effort-unit costs one Point). Consequently, the employer’s income is the higher the lower the wage payment and the more effort the assigned applicant eventually exerts. The applicant's income is the higher the higher the wage payment and the smaller the effort exerted. Because the applicant can freely set her effort level (if the employer does not impose a minimum effort level, see below), the unconditional wage payment can be interpreted as reflecting the employer’s level of trust towards the applicant (but see below). Accordingly, because there is no incentive to exert any effort, the exerted effort level can be interpreted as the applicant’s degree of (positive) reciprocity. This interaction-mechanism is based on the so-called `gift-exchange game´ [36], a workhorse commonly used in experimental economics for stylized employer-employee relationships. In the present setup, 1 Point was worth 1 NIS (Israel; West Bank); 0.18 Euro (Germany). Participants who on average paid higher wages to compatriots (same sex applicants) than to foreigners (opposite sex applicants) were classified as displaying out-group discrimination, whereas those who showed the opposite pattern were classified as portraying in-group discrimination. The rest were considered neutral with regard to wages.

#### Imposition of a minimum effort level

In conjunction with wage payment, employers were offered the costless opportunity to impose a fixed minimum effort level of 10 Units on each applicant, which restricted the applicant’s effort choice to 10–120 (the applicant could thus not choose an effort level below 10 Units; see also [37]). Imposing such a restriction on an applicant conveys that the employer does not trust the applicant to otherwise exert any effort. Participants who imposed a minimum effort level on foreign (opposite sex) applicants more often than on compatriots (same sex applicants) were classified as exhibiting out-group discrimination (note that the number of foreigners subject to an imposed minimum effort level was first divided by two; this was done because in the fixed set of twelve 12 résumés there were always eight foreigners and only four compatriots on which a minimum effort level could be imposed). Participants who showed the opposite pattern were classified as showing in-group discrimination, whereas the rest were considered neutral with regard to imposing a minimum effort level.

## Results

### Preliminary Analyses

Table 1 presents the descriptive statistics of our self-esteem questionnaire as well as the results of pair-wise *t*-tests comparing pre-decision self-esteem with post-decision self-esteem. These tests show that in both Germany and Israel, self-esteem levels decreased right after the employment task.

Because our discrimination measures were categorical, we used χ² to pair-wise investigate the associations among the different discrimination indices. Regarding ethnicity-based discrimination, the strongest association was found between rank-order and wages (φ_c_ = .36, *p* < .001; as to interpreting the effect size, φ_c_ can be considered a point-biserial correlation coefficient [38]). Concerning sex-based discrimination, the strongest association was found also between rank-order and wages (φ_c_ = .25, *p* < .001). Pertaining to the association between ethnicity- and sex-based discrimination, the strongest association was found between rank-ordering with regard to ethnicity and rank-ordering with regard to sex (φ_c_ = .24, *p* < .01). Because the associations were consistently weak, and the three different indices were theoretically distinct, we kept them separate for all analyses.

### Ethnicity-Based Discrimination

Regarding ethnicity-based discrimination, the descriptive results are summarized in the first three columns of Table 2. All three discrimination indices (i.e., rank-order of applicants-match, wage payment, imposition of minimum effort level) suggest that participants consistently either favored their compatriots or were impartial (note that if the participants are on average impartial, they should pick an in-group member as their first choice only one third of the time, because there were twice as many foreigners as compatriots to pick from—this pattern can be observed in the Israeli data). The summary statistics across regions reveal out-group discrimination with regard to ranking and wage, and impartiality with regard to imposing a minimum effort level.

**Table 2:** Ethnicity-based discrimination: Frequency of discrimination, and changes in self-esteem according to discrimination.

	Number of participants displaying discrimination			Change in self-esteem		
	Out-group discrimination	Impartial	In-group discrimination	Out-group discrimination	Impartial	In-group discrimination
**Israel**						
**Match**	24	NA	44	-.15 (.47)	NA	-.21 (.51) **
**Wage**	30	15	23	-.21 (.52) *	-.07 (.42)	-.25 (.50) *
**Imposed minimum effort level**	11	37	20	.03 (.31)	-.16 (.48) *	-.33 (.57) *
**West Bank**						
**Match**	44	NA	24	.02 (.35)	NA	-.07 (.33)
**Wage**	35	12	21	.00 (.37)	.00 (.24)	-.06 (.34)
**Imposed minimum effort level**	17	42	9	.10 (.45)	-.03 (.20)	-.16 (.41)
**Germany**						
**Match**	37	NA	32	-.03 (.45)	NA	-.26 (.58) *
**Wage**	27	33	9	-.14 (.62)	-.16 (.47)	-.04 (.45)
**Imposed minimum effort level**	4	59	6	.08 (.42)	-.15 (.52) *	-.17 (.75)
**Summary**						
**Match**	105	NA	100	-.05 (.42)	NA	-.18 (.47) **
**Wage**	92	60	53	-.12 (.50) *	-.08 (.38)	-.12 (.35) *
**Imposed minimum effort level**	32	138	35	.07 (.39)	-.11 (.43) **	-.22 (.50) **

*Note*. Change in self-esteem was computed as the difference between pre-decision and post-decision self-esteem. Statistical significance levels were computed from paired samples *t*-tests.

** *p* < .01,

* *p* < .05

With regard to Hypothesis (1), according to which out-group discrimination would be expected to be positively associated with self-esteem, the evidence was quite supportive (the last three columns of Table 2 show changes in self-esteem according to discrimination; statistical significance was assessed with pair-wise *t*-tests). Concentrating on the averages computed across regions, ranking an out-group member as first choice was associated with decreased self-esteem, whereas picking a compatriot maintained initial levels of self-esteem (*t* = 2.09, *d* = .29, *p* < .05; to investigate differences in change scores, we used independent samples *t*-tests, and effect sizes were computed using means and standard deviations [39]). A similar pattern was observed for the imposition of a minimum effort level: those who discriminated against the out-group in imposing a minimum effort level did not experience a change in self-esteem, whereas both those who were impartial (*t* = 2.31, *d* = .44, *p* < .05 for the difference in self-esteem change between those who favored the in-group and those who were neutral) and those who favored the out-group experienced a drop in self-esteem (*t* = 2.66, *d* = .65, *p* < .01, for the difference in self-esteem change between those who favored the in-group and those who favored the out-group).

Regarding Hypothesis (2), we found no relation between pre-decision self-esteem and discrimination. Within and across regions, initial levels of self-esteem did not differ between participants who discriminated against compatriots, were impartial, or discriminated against foreigners (for all three discrimination indices, all *F* < 1).

### Sex-Based Discrimination

The data were collapsed across regions for analyses of sex-based discrimination. Because the means of the self-esteem scales varied slightly across regions (see Table 1), we centered the scores within regions before conducting the analyses (within region averages of pre-decision self-esteem were set to zero, and post-decision self-esteem was scaled accordingly). The descriptive statistics show that neither men nor women discriminated strongly against the other sex (first three columns of Table 3).

**Table 3:** Sex-based discrimination: Frequency of discrimination, and changes in self-esteem according to discrimination.

	Number of participants displaying discrimination			Change in self-esteem		
	Out-group discrimination	Impartial	In-group discrimination	Out-group discrimination	Impartial	In-group discrimination
Women						
Match	63	NA	40	-.16 (.55) *	NA	-.18 (.35) **
Wage	32	35	36	-.15 (.46)	-.09 (.37)	-.26 (.58) *
Imposed minimum effort level	18	78	7	-.09 (.58)	-.18 (.48) **	-.19 (.33)
Men						
Match	48	NA	54	.02 (.41)	NA	-.14 (.46) *
Wage	29	44	29	-.03 (.46)	-.08 (.52)	-.07 (.27)
Imposed minimum effort level	10	72	20	.10 (.35)	-.07 (.45)	-.12 (.45)
Summary						
Match	111	NA	94	-.07 (.48)	NA	-.16 (.41) **
Wage	61	79	65	-.09 (.46)	-.08 (.45)	-.17 (.43) **
Imposed minimum effort level	28	150	27	.00 (.47)	-.13 (.47) *	-.16 (.39) *

*Note*. Change in self-esteem was computed as the difference between pre-decision and post-decision self-esteem. Statistical significance levels were computed from paired samples *t*-tests.

** *p* < .01,

* *p* < .05

Concerning Hypothesis (1), the evidence was somewhat supportive (see last three columns of Table 3). The summary statistics show that participants who favored their own sex did not experience a change in self-esteem, whereas those who favored the out-group either in ranking or wages did experience such a drop. The results were similar for the imposition of a minimum effort level. However, the results were generally somewhat weaker than for ethnicity-based discrimination, and none of the changes in self-esteem differed statistically significantly between groups.

Regarding Hypothesis (2), we found that women who favored their own sex concerning wages initially had lower levels of self-esteem in comparison to women who favored men (difference in means. 30, *d* = .53, *p* = < .05), and there was a similar trend in comparison to women who were neutral (difference in means. 23, *d* = .40, *p* < .10). None of the other comparisons we ran to test Hypothesis (2) were statistically significant (all *F* < 1).

### Comparison of Ethnicity- and Sex-Based Discrimination

An anonymous reviewer suggested, besides separately investigating the effects of ethnicity- and sex-based discrimination, they should be analyzed simultaneously. This is, indeed, consistent with recent guidelines designed to remedy the currently hotly debated issue of false positives in psychology—results should be reported with and without other predictor variables [40].

To investigate whether ethnicity- and sex-based discrimination retain their predictive power when entered simultaneously, we ran a series of general linear models on the variable self-esteem change (*M* = -.14, *SD* = .71), which was constructed as the difference between post-decision and pre-decision self-esteem. Now that the data were collapsed, employers’ sex and ethnicity were controlled for in all the analyses reported on below (see Table 4). Although sex showed evidence of being associated with change in self-esteem when entered together with the two variables reflecting ethnicity- and sex-based discrimination in the rank-ordering of applicants, the association between sex and change in self-esteem disappeared when sex was entered into the general linear model either alone or together with only ethnicity (*p*s > .10). An association that emerges only if certain other variables are included in the analysis is not likely to be robust [40], and we therefore ignored this association. By contrast, all of the results regarding ethnicity- and sex-based discrimination remained virtually unchanged regardless of whether ethnicity or sex was controlled for.

**Table 4:** General Linear Models Predicting Change in Self-Esteem.

	Model 1			Model 2			Model 3		
	DF	SS	F	DF	SS	F	DF	SS	F
Model	5	3.14	2.99*	7	3.78	2.59*			
Error	196	41.20		194	40.56				
Background variables									
Ethnicity	2	.57	1.36	2	.84	2.01	2	1.31	3.04
Sex	1	.93	4.43*	1	.61	2.92	1	.54	2.52
Rank-order Discrimination									
Ethnicity-Based	1	.94	4.46*						
Sex-Based	1	.16	.76						
Effort level Discrimination									
Ethnicity-Based				2	1.44	3.35*			
Sex-Based				2	.66	1.58			
Wage Discrimination									
Ethnicity-Based							2	.08	.19
Sex-Based							2	.60	1.40

*Note*. Ethnicity and sex-based discrimination refer to whether (a) the first choice among applicants was of the same ethnicity (gender), (b) a lower, similar, or higher minimum effort level was imposed upon applicants of the same ethnicity (gender), or (c), a lower, similar, or higher wage was paid to applicants of the same ethnicity (gender), in Models 1, 2, and 3 respectively.

** *p* < .01

* *p* < .05

Regarding the ranking of the applicants, when entered simultaneously, only ethnicity-based, but not sex-based, discrimination was associated with change in self-esteem (see Model 1 in Table 4). Those who did not favor their own ethnicity experienced a drop in self-esteem: average change in this group was-.18 (95% *CI* = -.27 –-.09), whereas it was-.04 (95% *CI* = -.13 –.04) among those who favored their own ethnicity.

Regarding the imposition of a minimum effort level on applicants, a similar pattern was observed. Only ethnicity-based discrimination, but not sex-based discrimination, was associated with a change in self-esteem (see Model 2 in Table 4). The self-esteem of those who favored their own ethnicity did not drop (mean change. 01 (95% *CI* = -.14 – .16)), whereas the self-esteem of those who favored the out-group clearly dropped (mean change-.24 (95% *CI* = -.38 – -.10); the difference between these two groups was statistically significant at *p* < .01). The self-esteem of those who favored neither the in-group nor the out-group suffered only a non-significant drop in self-esteem (mean change-.08 (95% *CI* = -.22 – .06)). They differed statistically significantly from those who favored the in-group (*p* < .05), but not from those who favored the out-group (*p* = .09).

Regarding wages, none of the variables predicted a change in self-esteem (see Model 3 in Table 4). This is not altogether surprising, given that the paired-samples comparisons shown in Tables 2 and 3 already suggest that rather similar changes occurred in self-esteem regardless of whether discrimination with regards to wages occurred. In sum, the results of these additional analyses suggest that in the present context, ethnicity-based discrimination was more strongly associated with the maintenance of self-esteem. Sex-based discrimination could not further contribute to the maintenance of self-esteem when ethnicity-based discrimination was controlled for.

## Discussion

The present results provided, in an employment context, some tentative support for the full and unqualified self-esteem hypothesis: Women with low self-esteem were more likely to assign higher wages to women than to men (Hypothesis (2) based on corollary (2)), and those women also maintained their initial (although low) levels of self-esteem (Hypothesis (1) based on corollary (1)). By contrast, women who were impartial or favored men experienced a drop in self-esteem. More general support was found for Hypothesis (1): both ethnicity- and sex-based discrimination of out-groups were related to the maintenance of initial levels of self-esteem. As argued below, only those groups arguably more prone to experience stereotype threat in the present setting (women, Israelis, Germans) showed a decrease in self-esteem. Therefore, the relation between self-esteem and discrimination could only be observed in these groups.

### Implications for the Self-Esteem Hypothesis

Zanna and Fazio [41] suggested a standard sequence to social psychological research literatures: after an initial wave of studies that establish and replicate an effect, most studies are designed to find the limits and boundary conditions of the effect. This sequence also describes the literature on the self-esteem hypothesis. For instance, status [42], interpersonal liking [43], fairness [27], and identification [21] have all been shown to influence relations between discrimination and self-esteem. But at some stage, the standing of the initial self-esteem hypothesis has itself become uncertain and controversial. The vigilant search for the exact conditions under which discrimination is related to self-esteem has arguably identified so stringent boundary conditions for the phenomenon that critiques may be fully justified to ask whether the phenomenon exists at all [29]. Rather than search for the boundary conditions of the phenomenon, the present research attempted to investigate its existence in a meaningful and natural context.

Concerning the full and unqualified self-esteem hypothesis, according to which low or threatened self-esteem motivates intergroup discrimination, which in turn enhances self-esteem, we found some tentative support. Women with lower levels of self-esteem discriminated against men in wage payment and such discrimination was associated with women’s maintaining their initial levels of self-esteem. Although the effect was only found for women, and only for wage payment, it should be noted that women, not men, are generally discriminated against in the labor market, and that women are well aware of sex-discrimination in wages [44]. Thus, setting higher wages for women could have been a form of competitive discrimination that operated against intergroup norms by bringing about social change with reference to positive in-group distinctiveness. And this is exactly the type of situation in which the self-esteem hypothesis was originally meant to apply [24]. By contrast, discrimination of women regarding wage payment is more or less simply the rule, and such discrimination may therefore not have served the self-esteem of men.

Although the evidence for the full self-esteem hypothesis was ambiguous, we interpret the present results as rather firmly supporting corollary (1). The effect sizes were generally somewhat stronger for ethnicity-based discrimination, and sex-based discrimination was not associated with the maintenance of self-esteem when ethnicity-based discrimination was controlled for. Both ranking a compatriot as first choice and imposing a minimum effort level on compatriots were associated with the maintenance of self-esteem. However, it is noteworthy that such discrimination was not associated with an increase in self-esteem, but rather with maintaining self-esteem at its original level. As was suggested by Lemyre and Smith [15], discrimination may only protect self-esteem that would otherwise be lowered (also [16], [17]).

But why did the self-esteem of some of our participants decrease during the employment task? The results of Branscombe and Wann [21] suggest that stereotype threat could be relevant in this respect; specifically, they showed that discrimination functions to restore decreased self-esteem after stereotype threat. Stereotype threat refers to the experience of uncertainty or anxiety in a situation where a person has the potential to confirm a negative stereotype about their social group (for a recent review, see [45]). Besides depressing test performance (the most famous effect of stereotype threat), stereotype threat also decreases self-esteem [46]. It seems conceivable that our Israeli, Palestinian, and German participants, when faced with the task of deciding whether or not to favor one group over another, experienced stereotype threat in varying degrees. In this particular constellation of groups, Israel’s present presence in the West Bank, and the past persecution of Jews in Nazi Germany [47], could render Israelis and Germans, respectively, particularly prone to fear of being personally reduced to a negative stereotype (for accounts of contemporary collective guilt in Israel and Germany, see [47], [48]). Indeed, both Israelis’ and Germans’ self-esteem decreased during the employment task. In contrast, Palestinians may have felt less threatened by the task of comparing their compatriots with Israelis or Germans. Indeed, Palestinians self-esteem levels did not drop during the task. This can explain why the self-esteem hypothesis was not supported in the group of Palestinians.

The above discussed result regarding sex-based discrimination could also be explained from the perspective of stereotype threat. In an employment setting, women are likely to encounter more threatening stereotypes than men (e.g., warm but not particularly competent housewives, competent Ice Queens lacking warmth [49]). Indeed, only women’s self-esteem decreased during the task, which meant that only women could maintain their self-esteem through discrimination. The result that discrimination only relates to the maintenance, but not to the increase, of threatened self-esteem is also consistent with research on self-image maintenance that indicates that the motive behind derogation and stereotyping of out-groups is to maintain rather than maximize self-evaluation [50], [51].

Our results can be considered surprisingly supportive of the self-esteem hypothesis. However, there were important differences between the present study and previous studies. For instance, to the best of our knowledge, no previous study conducted with natural groups has tested both corollaries of the self-esteem hypothesis using a behavioral measure. Much of the controversy surrounding the self-esteem hypothesis has without doubt been due to the extremely diverse array of measures used to measure self-esteem. As noted by Rubin and Hewstone [7], one of the reasons for the inconsistencies in the empirical evidence is that researchers have tended to measure trait self-esteem, which is usually thought to be stable over long periods of time. Rather, the self-esteem hypothesis should be tested using measures of state self-esteem, because these assess the continually updated here-and-now experience of self-worth. Another important distinction is between personal and social self-esteem, the latter redefining self-esteem to refer to the esteem in which one holds one’s group. Although generally recommending that a social state self-esteem scale be used in future research, Rubin and Hewstone [7] advised against their blatant use, because they are likely to increase demand characteristics (participants are likely to note a connection between an item such as “I feel good about my group” and their behavior towards the in-group). Instead of the social state self-esteem scale, we therefore opted for a personal state self-esteem scale. Although this could be interpreted as a reductionist move [52] earlier work on social identity theory ([11], [16], [53], [54]) provides a theoretical underpinning for the belief that the motivation to enhance not only social but also personal self-esteem can account for intergroup discrimination [7]. Furthermore, the empirical evidence suggests that regarding their relations to in-group bias, personal and social self-esteem do not differ (in fact, the evidence appears to slightly favor the use of personal self-esteem scales; for a meta-analysis, see [55]). For these reasons, we do not see our reliance on a measure of personal rather than social self-esteem as a serious limitation. But we do acknowledge that implicit measures of social state self-esteem may be a useful addition to future research.

Regarding the strength of the phenomenon, the effects that we did reliably establish ranged from medium (picking a compatriot as first choice in the ranking task) to large (in-group vs. out-group discrimination when imposing the minimum effort level), in comparison to other social psychological effects [56]. This suggests that small effects may have gone unnoticed due to lack of statistical power. The small sample sizes were more generally a limitation of the present research, because they did not allow us to investigate the possible effect of the out-group in ethnicity-based discrimination (e.g., Israelis discriminating against Palestinians vs. Israelis discriminating against Germans).

### Ecological Validity

The vast majority of the research conducted with natural groups has relied on asking participants to provide questionnaire ratings of the out-group (e.g., of the 35 real group studies presented by Rubin and Hewstone ([7], Table 3) only two required participants to allocate points between themselves and the out-group). This is understandable, given the effort and cost involved in having participants from meaningful groups interact with each other on behavioral tasks. But is even the dictator-like allocation of points akin to real-world behavior? In contrast to point allocation, the stylized employment task used in the present study should do somewhat better with regards to ecological validity the major differences being that the relationship participants entered into was bilateral and real—the other person was real, the information was real, and the provided incentives were real. Currently, in the field of experimental economics, monetary incentives are a crucial argument for allowing generalizations from laboratory experiments to contexts outside of the laboratory: monetary incentives ensure that participants perceive their behavior as relevant, experience real emotions, and take decisions with real economic consequences [57] (for empirical evidence, see [58]).

Besides monetary incentives, we believe that the bilateral structure of the task further served to increase its realism. Because the employers expected to receive something from their applicants, trust considerations are likely to have been important. Trust, having been described as the “lubricant that enables most essential everyday decisions” ([59], p. 357), is not only essential in everyday life, but acquires special importance in the employer-employee relationships. Indeed, the ethic of mutual obligation inherent in the employer-employee relationship has been described as a gift exchange game: Hard work and loyalty are exchanged for job security and high wages. Trust is a constitutive mechanism in every bilateral employer-employee relationship, because the employer is neither able to agree ex ante on the applicant’s actions by contract nor to observe and pay or sanction all associated actions [60]. We thus believe that the importance of trust considerations, both in the laboratory experiment, and in real personnel selection, further increases the likelihood that our results from the laboratory may generalize to employment contexts outside of the laboratory.

An important concession that we made in order to increase ecological validity was to make several dimensions of categorization (ethnicity, sex, competence) relevant to the participants. Typically, when more than one dimension of categorization is made salient to people, intergroup bias is reduced relative to single categorization [61]. However, because real-life applicants will differ from each other on multiple dimensions, it was important to show that the explanatory power of the self-esteem hypothesis is not confined to single categorization situations. In fact, we also tested for double in-group and double out-group effects, but the results did not differ from those reported for single in-groups and out-groups.

Finally, and perhaps most importantly from the perspective of ecological validity, out-group discrimination with regards to résumé-based pre-selection—the type of discrimination reported on here—has also been documented in real-world settings [4]. We thus believe that the same psychological mechanism that caused discrimination in the present study is at least to some extent also responsible for it in real employer-applicant relationships.

It is important to note that we found some support for the self-esteem hypothesis using all three discrimination indices. Considering that critiques have suggested that the processes identified by the social identity approach may only apply in the positive domain [29], and real-world discrimination may often occur in the negative domain, it is important that some support for corollary (1) of the self-esteem hypothesis was found for all measures of discrimination. Also important is that whether or not there was a neutral or impartial option present did not systematically influence the results. Based on prior results, one could have expected that discrimination would be more strongly related to elevated self-esteem in the absence of an impartial or fair option (in-group bias could violate the fairness considerations made salient by such an option and thereby depress self-esteem [27]).

### Relation to Behavioral Economics Literature on Social Identity and Discrimination

Besides contributing to the social psychology literature, our work speaks to the research on identity-dependent behavior that has been carried out in the emerging field of behavioral economics. A central tenet of this research has been that identity (or self-image) processes are central to understanding individuals’ preferences regarding economic outcomes [62–69]. Consistent with this line of research, our results revealed that making decisions which affect economic outcomes has implications for the self-esteem of the decision-makers. Our results highlight the importance of considering potential differences in gender norms when assessing the implications of economic behavior for identity processes—the same behavior may have different meanings across genders, and this could, at least in some instances, explain observed gender differences (for a literature review on gender differences in preferences, see [70], also [71], [72]).

Our work also intersects with a series of experimental findings on ethnic- and sex-based discrimination in selection and allocation tasks [1], [33], [73]. In light of our results, the pursuit of self-esteem could provide a plausible psychological mechanism for explaining some of the distinct behaviors towards in- and out-group members that have been observed in various types of economic situations. The conditions under which self-esteem is relevant in various selection and allocation tasks that allow for intergroup discrimination should be investigated in future research.

### Implications for Personnel Employment

With regard to the present employment context, in which both ethnicity-based and sex-based discrimination are prevalent [1], [5], [6], our results can be considered quite promising because they illuminate at least one process that relates to discrimination. Although there is a vast amount of research that verifies the existence of discrimination towards low-status groups, most of that research has focused on demographic properties of the applicant. But there is comparatively little research on the recruiter side, and the studies that do exist have tended to focus on individual difference variables (e.g., high social dominance orientation is related to discrimination against low status groups [74]). By contrast, the present results suggest a more general mechanism potentially causing discrimination, and may help companies develop compensatory organization-level strategies to reduce the display of discrimination.

Some recommendations can be made based on the present results. For instance, applicants should be asked to provide anonymous résumés with no ethnic or gender information, or at least not photographs because these make such categorical information more salient [75]. But, more intriguingly, what about the ethnicity and gender of the people making the recruitment decisions? As discrimination does happen, as also documented by the present results, it would be safe to suggest that the people in charge of recruitment decisions should come from various ethnic backgrounds, and that half of them should be women. However, the results also suggested that it was those groups who, in the particular intergroup context of the present study, were likely to experience stereotype threat and thereby decreased self-esteem, that were most likely to discriminate. One implication of this dynamic could be that the people making employment decisions should themselves belong to groups least likely to experience stereotype threat, alleviating the need for self-esteem protection through discrimination. But even members of prototypical groups, such as white middle-class males, are likely to experience stereotype threat under some circumstances. Therefore, rather than focus on characteristics of the decision makers, more sophisticated methods directed towards extinguishing the self-esteem maintaining function of discrimination should be investigated. For instance, the results presented by Scheepers et al. [27] suggest that when intergroup fairness is primed, discrimination of the out-group will not lead to an increase in self-esteem. Perhaps emphasizing the competitive nature of the recruitment process would prime fairness concerns, thus negating the self-esteem maintaining function of discrimination.

### Limitations and Conclusions

Besides the above discussed limitations associated with ecological validity, our measure of self-esteem may also raise some concerns. First off, we decided to keep the measure short (three items) in order to save participants’ time, to alleviate the possible effects of boredom, such as careless or random responding, and to minimize potential spillover effects to the subsequent tasks. Furthermore, even still briefer measures of self-esteem have shown high validity. For instance, the Single-Item Self-Esteem Scale (SISE), which consists of only one item, has almost as high associations with various validity criteria, such as peer ratings of group behavior and academic outcomes, as do much longer and more thoroughly validated measures [76]. As another point of concern, our self-esteem scale is open to the same criticism as all other Likert scales. For instance, the difference between two levels of an ordinal scale cannot be assumed to be the same as the difference between two other levels, and, even more generally, there is no guarantee that such scales can be meaningfully compared across subjects—we do not know whether subjects interpret the intervals similarly. Because of such shortcomings, an objective measure of self-esteem, based, for instance, on a monetary mechanism, would have been a useful addition to our battery of tests. However, increasing our confidence in the results that we report on, parametric statistics, such as those that we employed, have been shown to be highly robust with respect to the violation of the assumption that the intervals between numbers have the same interpretation (for a review, see [77]).

In conclusion, the results of the present study may serve to alleviate some of the general feeling of “mismatch between the big claims made in the (social identity) theory and the methods being used to them, as though high-level intergroup conflict could be explained by one minimal group at a time” ([18], p. 213). The present results suggest that the self-esteem hypothesis of the social identity approach has at least some explanatory power when attempting to understand real-world intergroup conflicts. However, we also acknowledge that other theoretical perspectives that have accommodated previous results obtained within the social identity approach could also account for the present results (e.g., the sociometer theory of self-esteem argues that in-group favoritism and out-group hostility have positive implications for self-esteem because they are one way to promote one’s value to other group members and strengthen one’s position within a group [78]). Therefore, perhaps even more important than the theoretical contributions of the present research is that we provide novel data on the impact of the self-esteem hypothesis in a real-world context in which discrimination is known to occur. By doing so, we hope to provide a foundation on which to build interventions to alleviate such discrimination.
